# One-year results of the intravitreal administration of faricimab in patients with naïve type 3 macular neovascularization

**DOI:** 10.1186/s40942-026-00812-7

**Published:** 2026-02-09

**Authors:** Masaaki Saito, Kimihiro Imaizumi

**Affiliations:** 1 Department of Regional Vision Reconstruction, Fukushima Medical University, 1 Hikarigaoka, Fukushima, 960-1295 Japan; 2 Iwaki City Medical Center, Iwaki, Japan

**Keywords:** Type 3 macular neovascularization, Faricimab, Retinal angiomatous proliferation, Optical coherence tomography angiography, Age-related macular degeneration

## Abstract

**Background:**

To clarify the efficacy of the intravitreal injections of faricimab and the change in vascular morphology of retinal-retinal anastomosis (RRA) after treatment in patients with type 3 macular neovascularization (MNV).

**Methods:**

We retrospectively reviewed 20 consecutive eyes with treatment-naive type 3 MNV in 14 Japanese patients (mean age, 81.8 years) at different stages. All patients were treated with 3 or 4 consecutive monthly intravitreal injections of faricimab and then they were followed by a “treat and extend, then fix” regimen in the maintenance phase over a 12-month follow-up.

**Results:**

The mean logarithm of the minimum angle of resolution best-corrected visual acuity (BCVA) levels improved significantly (*P* < 0.01) from 0.87 at baseline to 0.64 at 12 months. Optical coherence tomography angiography (OCTA) findings confirmed RRA in 16 (84.2%) of the 19 eyes, except for the eye with stage 1 at baseline, and 15 (93.8%) of the 16 eyes demonstrated the presence of vascular continuity at 12 months. No progression of retinal pigment epithelium (RPE) atrophy was observed in any of the 3 eyes with RPE atrophy at baseline, and newly developed RPE atrophy was seen in 4 (23.5%) of the 17 eyes without RPE atrophy at baseline. All 20 eyes had dry macula and showed no other complications at 12 months.

**Conclusions:**

Intravitreal faricimab injections significantly improved VA, achieved dry macula, and nearly normalized the vessels on the RRA in most patients with type 3 MNV during the 12-month study period.

## Background

Type 3 macular neovascularization (MNV) is a subtype of exudative age-related macular degeneration (AMD) that was first described as retinal angiomatous proliferation (RAP) by Yannuzzi et al. [1] in 2001. Type 3 MNV has 4 stages of differentiation characterized by clinical and angiographic features [2]. Although the prevalence of type 3 MNV is low among Japanese patients with AMD relative to Caucasian patients, the natural course of type 3 MNV is well known to be associated with poor visual outcomes in comparison to typical exudative AMD [3–6].

Intravitreal anti-vascular endothelial growth factor (VEGF) therapy using bevacizumab (off-label), ranibizumab, aflibercept, or brolucizumab for patients with AMD is considered a major, first-choice, reasonable, and useful treatment, based on evidence of the relationship between VEGF and MNV complexes in vitro or in clinical trials [7–15]. Although anti-VEGF monotherapy for patients with type 3 MNV has been reported to be effective, some reports have shown limited efficacy [16–24]. The development of retinal pigment epithelium (RPE) atrophy is the most significant complication in patients with RAP either after treatment or on a natural course, and it can lead to a decline in visual acuity (VA) [25–28]. RPE atrophy has been reported to be related to the presence of reticular pseudodrusen (RPD) [26–32].

Retinal-retinal anastomosis (RRA) is an important diagnostic indicator of RAP lesions.^1,2^ We reported the importance of complete occlusion of the RRA in patients with RAP to prevent the recurrence of RAP lesions during combined therapy with anti-VEGF drugs and photodynamic therapy (PDT) [28, 32–36]. However, RPE atrophy develops after treatment in patients with type 3 MNV, despite achieving complete occlusion of the RRA [28]. In the treatment of patients with RAP, the prevention of RPE atrophy may be more important than achieving complete occlusion of the RRA to stabilize the VA as much as possible.

A new anti-VEGF drug, faricimab, has a unique mechanism that inhibits both VEGF-A and Ang-2, which can result in an interval of up to 16 weeks between the injection and stabilization of vessels [37]. It could potentially improve the outcomes more than previous reports if the intravitreal injection of faricimab could normalize the retinal vessels of RRA with a reduction in RAP lesions.

The purpose of the current study was to clarify the efficacy of intravitreal injections of faricimab and the changes in the vascular morphology of RRA after treatment in patients with type 3 MNV.

## Methods

### Study design and population

This study was a retrospective single-center chart review. We retrospectively reviewed 20 consecutive eyes from 14 Japanese patients with treatment-naive type 3 MNV at different stages. All patients provided their written informed consent for treatment, and the retrospective chart review of this study after the potential risks and benefits were explained in detail. This observational study on the treatment and follow-up of AMD and similar diseases, as well as the retrospective comparative analysis conducted in this study, were approved by the Institutional Review Board and Ethics Committee of Iwaki City Medical Center.

### Inclusion and exclusion criteria

Eligible patients were at least 50 years of age and had treatment-naïve type 3 MNV. The clinical diagnosis of type 3 MNV was established based on the identification of RRA on early phase FA or ICGA and the identification of a hotspot on late-phase ICGA [1, 2]. We also classified cases of type 3 MNV into four stages [2]. The exclusion criteria were previous treatment for RAP, tears in the RPE, and maculopathies, such as diabetic maculopathy, retinal vascular occlusion, or idiopathic macular telangiectasia.

### Treatment protocol

All patients were treated with 3 or 4 consecutive monthly intravitreal injections of faricimab, followed by 8 to 12 weeks interval fixed injections at the most appropriate interval in the maintenance phase. We decided the interval of fixed injection with “treat and extend, then fix” regimen as we reported, by extending it for 4 weeks or shortening it by 2 weeks according to the presence of intraretinal or subretinal fluid on OCT B scans [16, 38–40]. Intravitreal faricimab was injected 3.5 to 4.0 millimeters posterior to the corneal limbus into the vitreous cavity using a 30- or 34-gauge needle under topical anesthesia applied according to the Japanese guidelines. After 3 or 4 consecutive monthly intravitreal injections of faricimab, the drug was injected according to the original method of the “treat and extend, then fix” regimen.

### Imaging acquisition and analysis by two investigators

All patients underwent standardized examinations, including slit-lamp biomicroscopy with a contact lens, color or red-free fundus photography, fluorescein angiography (FA), indocyanine green angiography (ICGA) with a fundus camera (TRC-50 FA/IA/IMAGEnet H1024 system; Topcon, Tokyo, Japan), confocal scanning laser ophthalmoscopy (Mirante; Nidek, Gamagori, Japan), and spectral-domain optical coherence tomography (RS-3000, Mirante; Nidek, Gamagori, Japan) during the follow-up period. CRT and SFCT were measured at every visit using an internal caliper software program and Enhanced Depth Imaging method. The presence of RRA was detected using slit-lamp biomicroscopy with a contact lens, color or red-free fundus photography, and ICGA findings. OCTA (RS-3000, Mirante; Nidek, Gamagori, Japan) was also performed to determine whether RRA could be detected at baseline based on ICGA findings. After 12 months, the presence or absence of vascular continuity in the RRA detected at baseline was carefully examined using OCTA. RPE atrophy was defined as the area with an increased visibility of the choroidal vasculature on fundus photographs and SW-AF images with hypoautofluorescence spots greater than 175 μm, and it was measured manually [41]. We examined SW-AF at baseline and at month 12. Measurements were independently performed by two examiners, and the mean of the two values was calculated. Consistent results between the two examiners were defined as yes or no, whereas discrepancies were defined as unclear.

### Outcome measures

The primary efficacy endpoint was the change in BCVA from baseline to one year. BCVA was calculated using the logarithm of the minimum angle of resolution scale, using the best-corrected VA measured with a Japanese standard decimal VA chart. Secondary endpoints were changes in CRT, SFCT, the rate of RRA detected by OCTA findings, and the rate of progression of RPE atrophy or newly developing RPE atrophy from baseline to 1 year.

### Statistical analysis

A statistical analysis was performed using the Wilcoxon signed-rank test to analyze the visual, CRT, and SFCT outcomes (*P* < 0.05 were considered to indicate statistical significance).

## Results

All patients (2 men, 12 women; age range, 74–91 years; mean age ± standard deviation, 81.8 ± 5.3 years) were Japanese and were followed up for at least 12 months. The proportion of stage 3 MNV based on a previous report ^2^ was as follows: stage 1, 1 eye (5%); stage 2, 8 eyes (40%); stage 3, 8 eyes (40%); and stage 4, 3 eyes (15%) (Table 1). RPD was observed in all 20 eyes.

**Table 1:** Intravitreal faricimab for type 3 macular neovascularization

Case No.	Age (yrs)	Gender	Eye	Type 3 MNV Stage	VA (logMAR)	Baseline					VA (logMAR)	Month 12						
						CRT (µm)	SFCT (µm)	RRA (FA or ICGA)	RRA (OCTA)	RPD		CRT (µm)	SFCT (µm)	Dry macula	Normalization RRA by OCTA	No. Injections	Interval of injections (week)	Newly developing RPE atrophy
1	78	F	Left	2	0.40	294	149	Yes	Yes	Yes	0.30	143	79	Yes	Yes	6	12	No
2	77	F	Left	4	3.00	840	71	Yes	unclear	Yes	2.00	91	45	Yes	unclear	6	12	No
3	78	F	Right	3	0.30	408	248	Yes	Yes	Yes	0.52	101	192	Yes	Yes	6	12	No
4	80	M	Left	2	0.70	493	197	Yes	Yes	Yes	0.40	125	136	Yes	Yes	6	12	Yes
5	88	M	Right	2	1.10	643	101	Yes	Yes	Yes	0.22	147	62	Yes	Yes	6	12	No
6			Left	4	0.82	543	89	Yes	unclear	Yes	1.00	79	63	Yes	unclear	6	12	No
7	74	F	Right	2	0.70	225	107	Yes	Yes	Yes	0.40	137	87	Yes	Yes	6	12	Yes
8			Left	2	0.30	290	120	Yes	Yes	Yes	0.40	105	83	Yes	Yes	6	12	Yes
9	91	F	Right	3	1.10	463	188	Yes	Yes	Yes	0.40	126	96	Yes	Yes	6	12	No
10	77	F	Right	3	1.10	398	229	Yes	Yes	Yes	1.00	221	171	Yes	Yes	6	12	No
11			Left	3	1.10	440	210	Yes	Yes	Yes	1.00	150	133	Yes	Yes	6	12	Yes
12	83	F	Right	2	0.82	481	150	Yes	Yes	Yes	0.22	98	87	Yes	Yes	6	12	No
13			Left	2	1.00	288	140	Yes	Yes	Yes	0.52	158	93	Yes	Yes	6	12	No
14	85	F	Right	3	0.40	430	156	Yes	Yes	Yes	0.15	136	118	Yes	Yes	6	12	No
15	87	F	Right	2	1.30	897	177	Yes	unclear	Yes	1.05	225	88	Yes	unclear	6	12	No
16	79	F	Left	3	1.22	569	58	Yes	Yes	Yes	1.30	92	42	Yes	unclear	6	12	No
17	79	F	Right	1	0.00	210	232	No	NA	Yes	0.10	199	231	Yes	NA	6	12	No
18			Left	3	0.52	556	276	Yes	Yes	Yes	0.52	137	217	Yes	Yes	6	12	No
19	89	F	Right	3	0.52	472	121	Yes	Yes	Yes	0.52	150	100	Yes	Yes	6	12	No
20			Left	4	1.10	661	150	Yes	Yes	Yes	0.70	125	89	Yes	Yes	8	8	No
Mean	82				0.87	480	158				0.64	137	110			6.1	11.8	
SD	6.3					184	61					41	54			0.4	0.9	

F = female; M = male; MNV = macular neovascularization ; Month 12 = 12 months after treatment; VA = decimal visual acuity; CRT = central retinal thickness; SFCT =subfoveal choroidal thickness; RRA = retinal-retinal anastomosis; RPD = reticular pseudodrusen; OCTA = optical coherence tomography angiography; RPE = retinal pigmented epithelium; NA = not applicable; SD = standard deviation

**Fig. 1:**
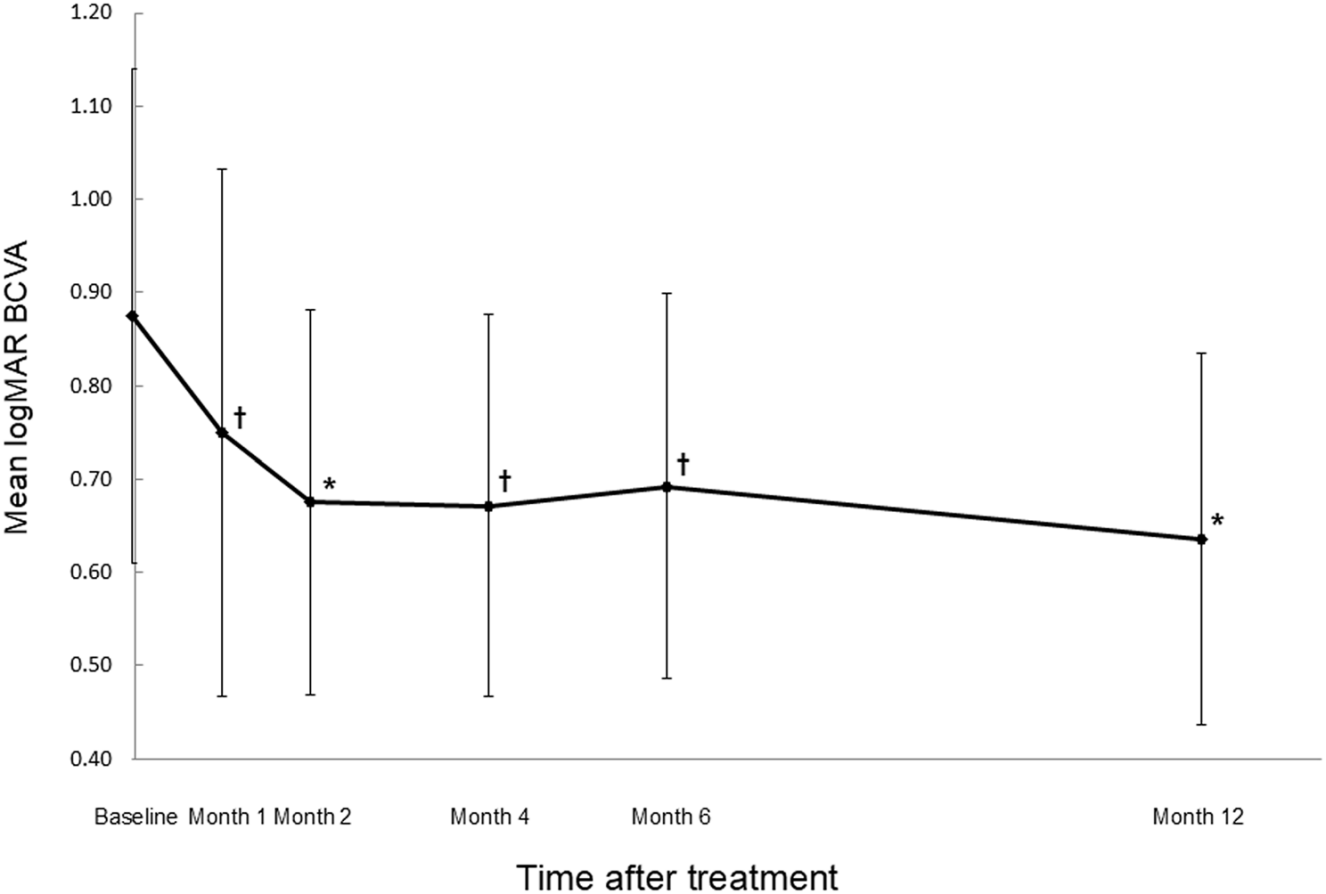
Changes in the mean best-corrected visual acuity (BCVA) in eyes with type 3 macular neovascularization treated with intravitreal faricimab injections during a 12-month period. The mean BCVA improved significantly after treatment. Months 1, 2, 4, 6 and 12 refer to the months after the initial treatment. Vertical bars are 95% confidence intervals of the means. ^†^*P* < 0.05; *P < 0.01 (Wilcoxon signed-rank test). logMAR, logarithm of the minimum angle of resolution

The mean logarithm of the minimum angle of resolution best-corrected visual acuity (BCVA) (Snellen equivalent) levels significantly improved from 0.87 (20/150) at baseline to 0.75 (20/112) at 1 month after treatment (month 1) (*P* < 0.05), 0.68 (20/95) at month 2 (*P* < 0.01), 0.67 (20/94) at month 4 (*P* < 0.05), 0.69 (20/99) at month 6 (*P* < 0.05) and 0.64 (20/87) at month 12 (*P* < 0.01) (Wilcoxon signed-rank test) (Fig. 1). The mean changes in BCVA at months 1, 2, 4, 6, and 12 were improvements of 1.25, 2.00, 2.03, 1.32 and 2.39 lines, respectively. At 12 months, an improvement in BCVA by three lines or more was seen in eight (40%) of the 20 eyes, and the remaining 12 (60%) eyes had stable BCVA (defined as a loss of fewer than three lines of vision). No eyes showed a decrease in BCVA of three or more lines during the 12 month follow-up period in the current study.

**Fig. 2:**
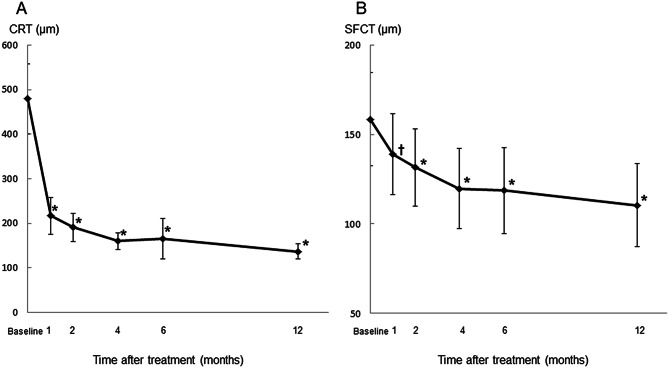
(**A**) Changes in the central retinal thickness (CRT) in eyes with type 3 macular neovascularization. The mean CRT significantly decreased after treatment. Months 1, 2, 4, 6 and 12 refer to the months after the initial treatment. Vertical bars are 95% confidence intervals of the means. *P < 0.0001 (Wilcoxon signed-rank test). (**B**) Changes in the subfoveal choroidal thinning (SFCT) in eyes with type 3 macular neovascularization. The mean SFCT significantly decreased after treatment. Months 1, 2, 4, 6 and 12 refer to the months after the initial treatment. Vertical bars are 95% confidence intervals of the means. ^†^*P* < 0.001; *P < 0.0001 (Wilcoxon signed-rank test)

The mean central retinal thickness (CRT) significantly decreased from 480 ± 184 μm at baseline to 217 ± 97 μm at month 1 (*P* < 0.01), 191 ± 74 μm at month 2 (*P* < 0.001), 160 ± 43 μm at month 4 (*P* < 0.0001), 165 ± 106 μm at month 6 (*P* < 0.0001), and 137 ± 41 μm at month 12 (*P* < 0.0001) (Wilcoxon signed-rank test) (Fig. 2a). The mean subfoveal choroidal thinning (SFCT) also significantly decreased to 139 ± 53 μm at month 1 (*P* < 0.001), 132 ± 50 μm at month 2 (*P* < 0.0001), 120 ± 52 μm at month 4 (*P* < 0.0001), 119 ± 56 μm at month 6 (*P* < 0.0001), and 110 ± 54 μm at month 12 (*P* < 0.0001) relative to baseline (158 ± 61 μm) (Wilcoxon signed-rank test)(Fig. 2b).

**Fig. 3:**
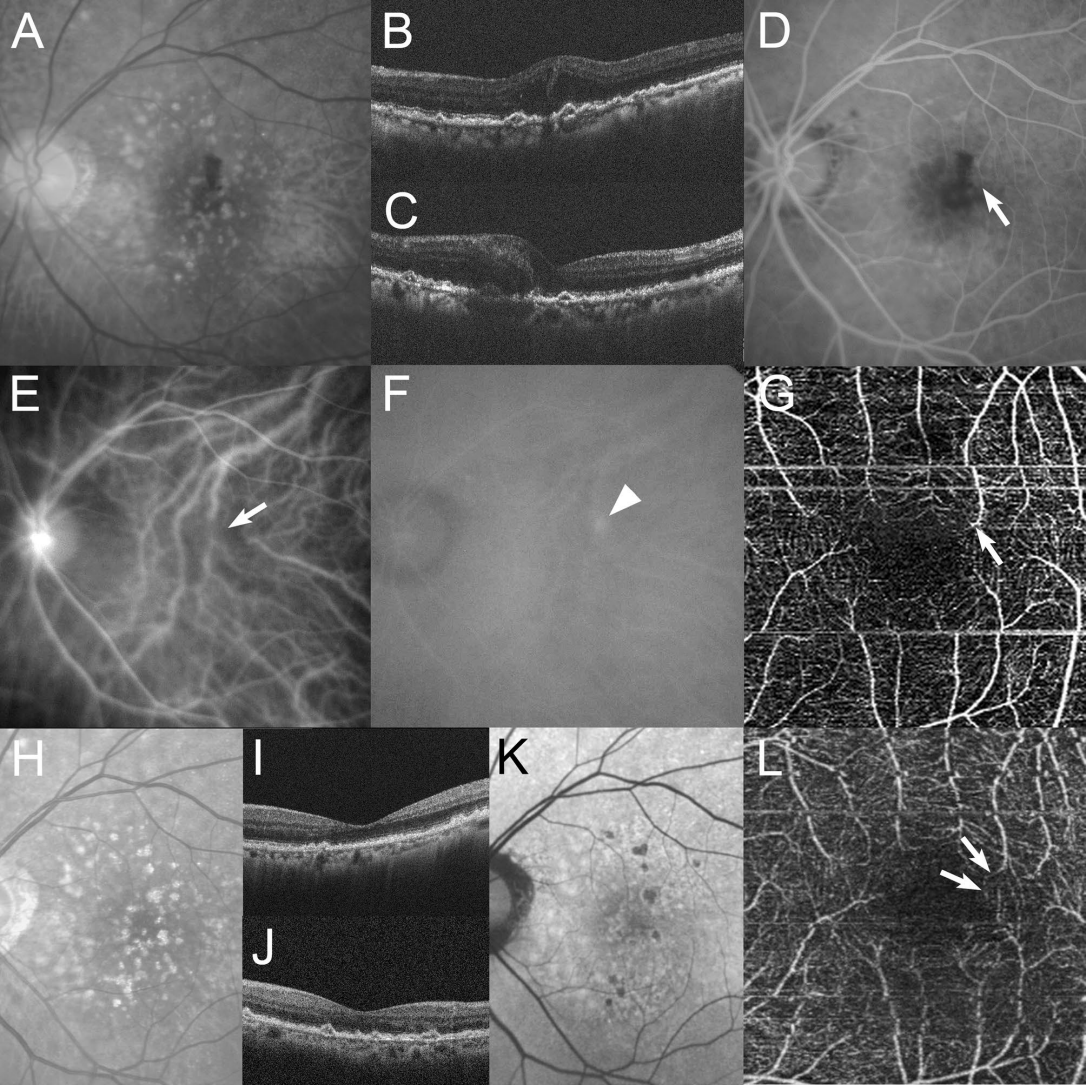
A 78-year-old woman with stage 2 type 3 macular neovascularization (MNV) (case 1 on the Table 1). At baseline, the best-corrected visual acuity (BCVA) is 0.40 logarithm of the minimum angle of resolution VA (Snellen equivalent; 20/50). **A** A red-free photograph shows intraretinal and preretinal hemorrhages, drusen and reticular pseudodrusen in the macular area. Horizontal (**B**) and vertical (**C**) optical coherence tomography (OCT) images show intraretinal fluid (IRF). **D** A fluorescein angiography image shows leakage due to MNV and the retinal-retinal anastomosis (RRA) (arrow). **E** An early-phase indocyanine green angiography (ICGA) image shows RRA (arrow). **F** A late-phase ICGA image shows a type 3 MNV lesion as a focal area of intense hyperfluorescence (hot spot) (arrowhead). **G** An en face image of OCT angiography demonstrates RRA shown, which is demonstrated by abnormal vessels with discontinuation and dipping (arrow). **H** At twelve months, after 6 faricimab injections, the BCVA slightly improved to 0.30 logarithm of the minimum angle of resolution VA (Snellen equivalent; 20/40). A red-free photograph shows no hemorrhages or edema in the macular area. Horizontal (**I**) and vertical (**J**) OCT images show the resolution of the IRF. **K** A short-wavelength autofluorescence image shows no retinal pigmented epithelium atrophy as hypoautofluorescence involving the fovea. **L** An en face image of OCT angiography demonstrates an improvement of the vessel discontinuation detected as retinal-retinal anastomosis at baseline (arrows)

**Fig. 4:**
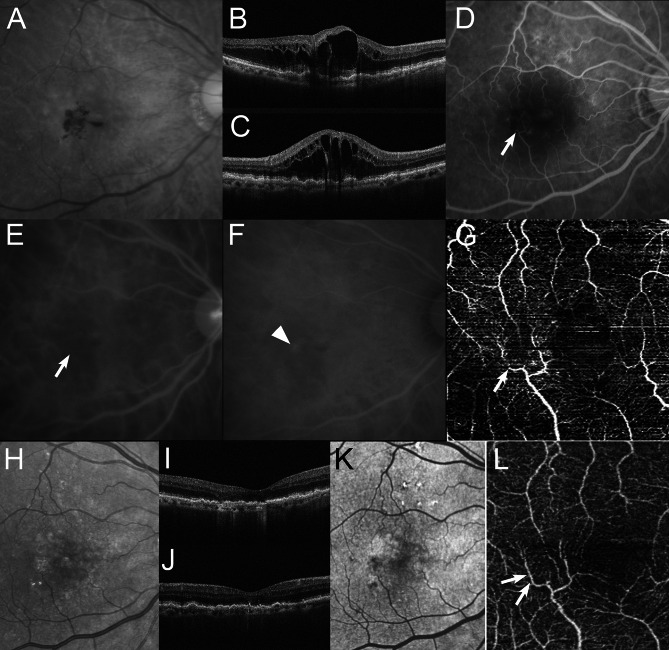
Aa 88-year-old man with stage 2 type 3 macular neovascularization (MNV) (Case 5 in Table 1). At baseline, the best-corrected visual acuity (BCVA) is 1.10 logarithm of the minimum angle of resolution VA (Snellen equivalent; 20/250). **A** A red-free photograph shows intraretinal and preretinal hemorrhages, intraretinal fluid (IRF), drusen and reticular pseudodrusen in the macular area. Horizontal (**B**) and vertical (**C**) optical coherence tomography (OCT) images show altitude IRF in the wide macular area. **D** An early-phase fluorescein angiography image clearly shows slight leakage due to MNV and retinal-retinal anastomosis (RRA) (arrow). Early-phase (**E)** and late-phase (**F)** indocyanine green angiography (ICGA) images show RRA (arrow) and a type 3 MNV lesion as a focal area of intense hyperfluorescence (hot spot) (arrowhead). **G** An en face image of OCT angiography clearly demonstrates the RRA, which is indicated by abnormal vessels with discontinuation and dipping (arrow). **H** At 12 months, after 6 faricimab injections, the BCVA improved to 0.40 logarithm of the minimum angle of resolution VA (Snellen equivalent; 20/33). A red-free photograph shows disappearance of both hemorrhages and edema in the macular area. Horizontal (**I**) and vertical (**J**) OCT images show the resolution of the IRF. **K** A short-wavelength autofluorescence image shows no retinal pigmented epithelium atrophy as hypoautofluorescence in the macular area. **L** An en face image of OCT angiography clearly demonstrates the improvement of vessel discontinuation detected as retinal-retinal anastomosis at baseline (arrows)

At baseline, all eyes had intraretinal fluid (IRF), 4 (20%) of the 20 eyes had subretinal fluid (SRF), and 11 (55%) of the 20 eyes had a retinal pigment epithelial detachment (PED). IRF resolved in all 20 (100%) eyes at a mean of 6.2 weeks after baseline. SRF resolved in all 4 (100%) eyes at a mean of 4.0 weeks after baseline. PED resolved in all 11 (100%) eyes at a mean of 8.0 weeks after baseline. None of the eyes showed recurrence of IFR, SRF, or PED, and dry macula was achieved at 12 months in all 20 eyes. The number of faricimab injections during the 12 months, including the initial regimen, was 6 in 19 (95%) of the 20 eyes. The interval between faricimab injections at 12 months was 12 weeks in all 19 eyes that received three consecutive monthly intravitreal injections. The remaining eye had residual IRF at 3 months and received four consecutive monthly injections of faricimab, followed by an 8-week interval of a fixed regimen, for a total of eight injections over 12 months. Figures 3 and 4 show the ocular images obtained from patients treated with faricimab.

**Fig. 5:**
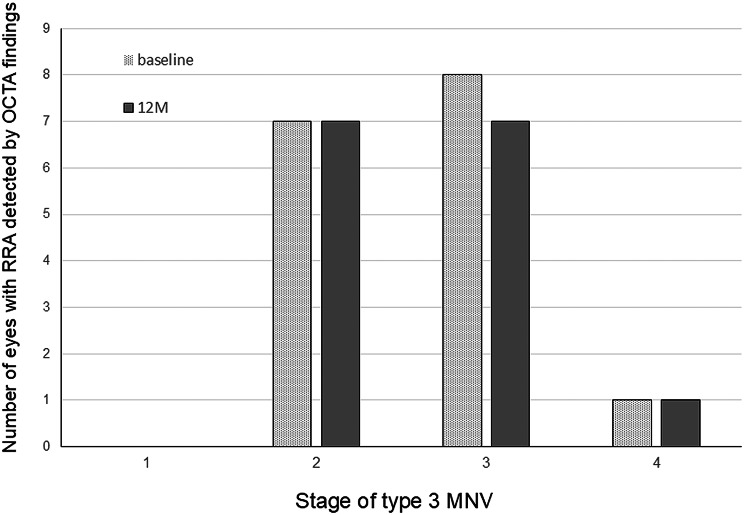
The correlation between the number of eyes with retinal-retinal anastomosis (RRA) detected by the optical coherence tomography angiography (OCTA) findings at baseline and 12 months and the stage of type 3 macular neovascularization (MNV)

At baseline, RRA was detected in all 19 eyes, except for eyes with stage 1 (Figs. 3 and 4). OCTA findings confirmed RRA in 16 (84.2%) of the 19 eyes (stage 2, 7 eyes; stage 3, 8 eyes; and stage 4, 1 eye) (Table 1) (Figs. 3, 4 and 5). The presence of RRA could not be determined in the remaining 3 eyes because of unclear OCTA findings. After treatment with faricimab, OCTA demonstrated that 15 (93.8%) of the 16 eyes showed improvement in discontinuation of RRA at baseline (stage 2, 7 eyes; stage 3, 7 eyes; and stage 4, 1 eye) (Table 1) (Figs. 3, 4 and 5). The remaining eyes showed unclear OCTA findings at 12 months.

RPE atrophy detected by short-wavelength autofluorescence (SW-AF) was observed in 3 (15%) eyes at baseline. At month 12, no progression of RPE atrophy was observed in any of the 3 eyes, and newly developed RPE atrophy was observed in 4 (23.5%) of the 17 eyes without RPE atrophy at baseline. All 4 eyes had RPE atrophy at the extra-fovea and showed improvement in BCVA with a mean of 1.51 lines at month 12.

There were no complications such as any unexpectedly increasing subretinal hemorrhage (> 1 disc diameter), RPE tear, ocular inflammation, increased intraocular pressure > 21 mmHg, severe visual loss, endophthalmitis, cataract progression, or systemic adverse events.

## Discussion

The current study showed that intravitreal faricimab injections significantly improved VA and the anatomical changes in patients with type 3 MNV during the 12 months of the study. In most cases (19 of the 20 eyes) received 12 weeks interval fixed injection was administered during the maintenance phase. Moreover, OCTA also demonstrated an improvement in the discontinuation of RRA at 12 months in 15 (93.8%) of the 16 eyes with RRA shown by OCTA at baseline.

Type 3 MNV has been reported to have a poor natural course and a tendency to be refractory to therapies relative to typical exudative AMD [3–6]. Anti-VEGF therapy has become an evidence-based treatment for AMD worldwide [7–15, 37]. Furthermore, it has been reported that type 3 MNV tends to respond better to anti-VEGF drugs than other types of MNV [22]. A systematic review study with meta-analysis for 34 studies showed a mean gain of 5.16 letters and 10.38 letters was shown in the anti-VEGF monotherapy group and combined with anti-VEGF and PDT group, respectively (anti-VEGF group vs. combined group, *p* < 0.01) [23]. The current study showed that BCVA at 12 months improved by a mean of 2.39 lines from baseline, which was slightly better than that reported in previous studies using combined therapy. Type 3 MNV, in some cases, could require intense and prolonged treatment due to a frequent recurrence of membrane activity [18, 19]. The SEVEN-UP Study showed a mean 8.6-letter decrease in BCVA during the 7-year follow-up with ranibizumab, with one of the main reasons being the persistence of active exudative disease with the PRN regimen [42]. In the current study, we used a “treat and extend, then fix” regimen, which may be easier for all investigators and less stressful for patients owing to the treatment schedule. Furthermore, all 20 eyes achieved dry macules with an average of 6.1 injections over 12 months. Although the average number of injections at 12 months in the current study was higher than the 4.9 injections in the review study, the interval between faricimab injections at 12 months was 12 weeks in 19 (95%) of the 20 eyes, thus allowing the 19 eyes to accumulate fewer injections in the second year. The current study using a “treat and extend, then fix” regimen may be effective in improving BCVA and achieving dry macula, and the results of further follow-up studies need to be considered.

Macular atrophy is well known to be an important factor impeding BCVA improvement in long-term follow-up [42], and type 3 MNV is also known to have a high prevalence of RPE atrophy after treatment [25–32]. Although we have reported the efficacy of combined therapy with anti-VEGF drugs and PDT, demonstrating that it is associated with less recurrence than anti-VEGF monotherapy, RPE atrophy cannot be prevented and should be related to the decline in VA, as described in previous reports [28]. However, theoretically, it is difficult to avoid macular atrophy. Although the follow-up period of the current study was only 12 months, no progression of RPE atrophy was observed in any of the 3 eyes with RPE atrophy at baseline. Moreover, newly developing RPE atrophy was seen in 4 (23.5%) of the 17 eyes without RPE atrophy at baseline and the 4 eyes had RPE atrophy at the extra-fovea and showed improvement in BCVA with a mean of 1.51 lines at month 12. Faricimab has a unique mechanism that inhibits both VEGF-A and Ang-2; however, its clinical efficacy remains unclear [37]. Although the correlation between Ang-2 inhibition and RPE atrophy is currently unclear, the fact that faricimab may reduce macular atrophy should be further considered in clinical practice.

Type 3 MNV has a unique characteristic of RRA, which has been considered important for diagnosing type 3 MNV and as a sign of recurrence [1, 2]. Miere et al. reported that OCTA reported that OCTA in treatment-naive type 3 neovascularization always showed a high-flow tuft abnormal outer retinal proliferation, frequently associated with a small clew-like lesion in the choriocapillaris layer [43]. We focused on the change in RRA on OCTA to examine the minimal vascular change as a possible indicator of the efficacy of Ang-2 inhibition. In this study, the OCTA findings confirmed RRA in 16 (84.2%) of the 19 eyes, except for the eye with stage 1 at baseline, and 15 (93.8%) of the 16 eyes demonstrated the presence of vascular continuity. Although these results should be examined with other anti-VEGF drugs, the improvement demonstrated by the OCTA findings may be attributed to the normalization and stabilization effects on the vessels, which we speculate is a result of Ang-2 inhibition by faricimab. Although the mechanism of vascular continuity of the RRA could not be clarified in this study, we speculated that the dipping vessel blood vessels of the RRA toward type 3 MNV, which is a characteristic of the RRA, might approach normalization due to the regression or dysfunction of type 3 MNV. The normalization of vessels may also prevent the development of RPE atrophy. Furthermore, the superiority of inhibiting both VEGF-A and Ang-2 may be strongly related to the fact that the 12-week interval was achieved in most of the 20 eyes (19 of 20 eyes) and that dry macula was observed in all 20 eyes at 12 months.

It is well known that in patients with unilateral RAP, the opposite eye is at a high risk of developing RAP lesions [41, 44, 45]. It is difficult to diagnose the signs of the onset of type 3 MNV lesions using the usual color fundus or OCT findings. We previously reported that abnormalities identified by SW-AF and near-infrared autofluorescence (NIR-AF) imaging may be important for predicting the imminent onset of RAP lesions [41]. An examination for capturing NIR-AF images could not be performed in the current study. Therefore, we defined the maximum interval as 12 weeks during the maintenance phase to prevent missing signs of RAP lesion onset. Moreover, the mean age of patients with RAP tends to be higher than that of patients with other types of AMD in Japan, which is better to prevent minimal changes in recurrence or newly developing RAP lesions in patients who are unaware of subjective symptoms [5, 6]. In the current study, 3 patients with unilateral RAP developed new RAP in the opposite eye on the scheduled visit date without a decline in VA. A maximum interval of 12 weeks may therefore be the most reasonable and permissible for patients with type 3 MNV.

During the 12-month follow-up period in the current study, no complications, such as intraocular inflammation (IOI), which can occasionally occur after brolucizumab injection, or other general complications, were observed following injections of faricimab. As reported previously, if IOI is expected or followed, the use of the laser flare-cell photometer (LFP) score may prove to be beneficial [46].

The present study is associated with several limitations. This was a single-center retrospective study with a small sample size and a follow-up period of 1 year. The small sample size may have limited the statistical analysis. Further large-scale, long-term prospective randomized trials are needed to clarify the efficacy, safety profile, and OCTA findings of intravitreal faricimab in patients with type 3 MNV.

## Conclusions

In conclusion, the present findings demonstrate that intravitreal faricimab injection is safe and effective for improving VA, achieving dry macula, and nearly normalizing the vessels on the RRA in most patients with type 3 MNV.

## Data Availability

The datasets provided within the manuscript and analyzed during the current study are available upon reasonable request from the corresponding author.

## Abbreviations

AMD: Age-related macular degeneration
BCVA: Best-corrected visual acuity
CRT: Central retinal thickness
FA: Fluorescein angiography
ICGA: Indocyanine green angiography
IRF: Intraretinal fluid
LogMAR: Logarithm of the minimum angle of resolution
MNV: Macular neovascularization
OCTA: Optical coherence tomography angiography
PED: Retinal pigment epithelial detachment
RAP: Retinal angiomatous proliferation
RPD: Reticular pseudodrusen
RPE: Retinal pigment epithelium
RRA: Retinal-retinal anastomosis
SFCT: Subfoveal choroidal thinning
SRF: Subretinal fluid
SW-AF: Short-wavelength autofluorescence
VA: Visual acuity
VEGF: Anti-vascular endothelial growth factor
